# Exploring the responses of smallscale poultry keepers to avian influenza regulations and guidance in the United Kingdom, with recommendations for improved biosecurity messaging

**DOI:** 10.1016/j.heliyon.2023.e19211

**Published:** 2023-08-17

**Authors:** Sarah Jewitt, Matthew Smallman-Raynor, Emma McClaughlin, Michael Clark, Stephen Dunham, Sol Elliott, Alastair Munro, Tamsin Parnell, Rachael Tarlinton

**Affiliations:** a School of Geography, University of Nottingham, UK; b School of English, University of Nottingham, UK; c One Virology, The Wolfson Centre for Global Virus Research, School of Veterinary Medicine and Science, University of Nottingham, UK

**Keywords:** Backyard poultry keepers, Behaviour settings theory, Biosecurity, Highly pathogenic avian influenza (HPAI), IBM-WASH, United Kingdom

## Abstract

Understanding how smallscale (‘backyard’) poultry keepers interpret and respond to governmental directives designed to reduce the transmission of highly pathogenic avian influenza (HPAI) is of paramount importance in preparing for future HPAI outbreaks. Qualitative insights from open questions in an online survey conducted during the 2021–22 HPAI season (1,559 responses) shed light on smallscale poultry keepers' understanding of, and responses to, governmental directives to control HPAI exposure and onwards transmission. A follow-up participatory workshop (21 participants) explored the HPAI-related information sources used by smallscale poultry keepers, their trust in these sources, perceptions of HPAI-related risk, and interpretation of, opinions on and adherence to government regulations and communications regarding biosecurity and housing measures. This paper draws on a multi-scale behaviour change model to explore barriers to compliance with HPAI-related regulations. Insights from behaviour settings theory reveal how poultry-keeping settings and routines might be ‘disrupted’ and ‘re-configured’ to improve long-term biosecurity and reduce the risk of HPAI exposure. The findings highlight the need for HPAI-related guidance that is tailored to smallscale poultry keepers. This guidance should include clear action points and simple, practical, affordable and sustainable suggestions for improving compliance with biosecurity measures.

## Highlights

• Smallscale poultry keepers' responses to avian influenza regulations are explored
• IBM-WASH and settings theory are applied to poultry keeping for the first time
• These approaches identified barriers and potential enablers to improving biosecurity
• Smallscale poultry keepers need tailored guidance on avian influenza prevention

## 1 Introduction

Highly pathogenic avian influenza (HPAI) is a devastating disease of poultry that causes rapid mortality in affected flocks and, due its zoonotic potential, has important implications for human health [1]. Outbreaks of HPAI due to influenza A/H5N1 in the United Kingdom (UK) and Europe have been linked to virus introductions by migratory wildfowl with onwards transmission to commercial and ‘backyard’ poultry holdings [[2], [3], [4], [5]].

The current H5N1 outbreak is unprecedented in the UK. The outbreak first came to notice in mid-October 2021 when the virus was detected among rescued wild swans at a sanctuary in Worcestershire [6]. Since then, the outbreak has been associated with over 340 documented HPAI events in commercial poultry, backyard flocks and other settings [7] whilst mass die-offs of wild birds have been widely reported [8]. To limit the spread of the disease in the first year of the outbreak, a UK-wide Avian Influenza Prevention Zone (AIPZ) was declared between November 3, 2021 and August 16, 2022. Additional ‘housing measures’ were introduced between November 29, 2021 and May 2, 2022 [9] that required “poultry and other captive birds to be housed or otherwise kept separate from wild birds.” Similar measures were implemented again from autumn 2022. These measures aimed to prevent the spread of HPAI from wild birds to commercial or backyard poultry and other captive birds whilst also seeking to promote their welfare, protect human health, minimise impacts on the poultry sector, the economy and international trade [10] and mitigate the risk of ‘spill back’ from infected premises to wild birds [11].

Drawing on experiences from the 2021–22 HPAI season in the UK, this study explores smallscale poultry keepers' understanding of, and responses to, governmental directives to control HPAI exposure and onwards transmission. The research is timely in its efforts to co-produce effective health communication and behaviour change approaches with the potential to reduce the risk of HPAI exposure and transmission to smallscale poultry flocks. It offers conceptual and methodological novelty whilst promoting ‘South-North’ knowledge transfers by adapting behaviour change approaches designed for low- and middle-income countries in the ‘global South’ to high-income contexts in the ‘global North.’ The study's significance lies in its potential to inform behaviour change communication designed for smallscale poultry keepers in high-income contexts and to identify simple, practical, and affordable ways for them to adopt and habitually improve biosecurity measures.

Until recently, the susceptibility of smallscale or ‘backyard’ flocks to HPAI and their role in onwards transmission were considered less important in Europe than Asia [5,12]. Although some European studies have investigated HPAI-related risks presented to commercial poultry farms by backyard flocks [5,13], smallscale keepers' knowledge, attitudes and implementation of appropriate biosecurity measures are poorly understood [14] and we are not aware of any UK-wide studies of these issues. With 33 outbreaks recorded in UK backyard flocks during the 2021–22 season [6,15], the need to understand HPAI transmission and biosecurity in backyard flocks in the UK – and other high-income contexts – has become increasingly apparent. Challenges to achieving this include the absence of an agreed definition of a ‘backyard keeper’ [12] plus a dearth of information on their numbers and characteristics. Unlike Northern Ireland (NI), where all flocks must be registered with the Department of Agriculture, Environment and Rural Affairs (DAERA), it is optional for keepers of fewer than 50 birds in England, Wales and Scotland to register their flocks with the Department for Environment, Food and Rural Affairs (Defra) and the Animal and Plant Health Agency (APHA). Poultry Registry data for 2021 reported a total of 40,260 premises (272,446,498 birds in total) of which 20,573 had fewer than 50 birds [16]. A 2020 survey estimated that there were 1,338,000 chicken owners in the UK [17] highlighting the difficulties (compared to the far more highly regulated commercial poultry sector) of gaining information on smallscale keepers and communicating HPAI-related information to them.

Similar obstacles are apparent in other high-income countries where a lack of information on backyard flocks and limited knowledge of poultry-related infectious diseases amongst their keepers hampers efforts to control outbreaks of HPAI and other highly transmissible diseases [[18], [19], [20], [21], [22], [23]]. Although there are an estimated 10 million backyard chicken owners in Europe, 7–13 million in the US, and 416,000 in Australia [20,24], requirements for poultry keepers to register or obtain permits for their flocks vary widely; often applying only to urban areas or larger flocks [[25], [26], [27], [28], [29], [30]].

## 2 Conceptual framework

Reflecting concerns about the human health risks presented by A/H5N1 in Asia [31], a significant body of work exists on Asian backyard poultry keepers’ knowledge and responses to HPAI-related risk. Much of this work indicates limited understanding or implementation of biosecurity measures [[32], [33], [34], [35], [36], [37], [38], [39]]. To address this, various information, communication and education approaches and behaviour change technologies/models have been utilised in interventions seeking to reduce HPAI-related human health risks from backyard poultry [34,[36], [37], [38],[40], [41], [42]].

Environmental health-related interventions in low- and middle-income contexts are often underpinned by behaviour change theory. This helps to identify behavioural determinants likely to stimulate a desired change although the focus has often been at the individual scale [43,44]. In developing their Integrated Behavioural Model for Water, Sanitation, and Hygiene (IBM-WASH), Dreibelbis et al. [45] sought to encompass multi-scale behavioural influences across five levels and three dimensions (Table 1). The three dimensions capture ‘contextual’ influences that characterise intervention settings, ‘psychosocial’ factors that include behavioural determinants related to opportunity, ability and motivation, and ‘technology’ characteristics where this forms part of an intervention. Intersecting these are four levels (structural/societal, community, household and individual). A fifth ‘habitual’ level focuses on factors influencing sustained behaviour change in view of the public/environmental health implications of ‘backsliding’ to previous behaviours [[45], [46], [47], [48]].

**Table 1:** The integrated behavioural model for water, sanitation, and hygiene (IBM-WASH).

Level	Contextual factors	Psychosocial factors	Technology factors
Societal/Structural	Policy and regulations, climate and geography	Leadership/advocacy, cultural identity	Manufacturing, financing, and distribution of the product; current and past national policies and promotion of products
Community	Access to markets, access to resources, built and physical environment	Shared values, collective efficacy, social integration, stigma	Location, access, availability, individual vs. collective ownership/access, and maintenance of the product
Household/interpersonal	Roles and responsibilities, household structure, division of labour, available space	Injunctive norms, descriptive norms, aspirations, shame, nurture	Sharing of access to product, modelling/ demonstration of use of product
Individual	Wealth, age, education, gender, livelihoods, employment	Self-efficacy, knowledge, disgust, perceived threat	Perceived cost, value, convenience, and other strengths and weaknesses of the product
Habitual	Favourable environment for habit formation, opportunity for and barriers to repetition of behaviour	Existing water and sanitation habits, outcome expectations	Ease/effectiveness of routine use of product

Complementing the IBM-WASH model's habitual level, Curtis and colleagues' ‘behaviour settings theory’ [49] builds on ideas of ‘social practice’ [50] to emphasise how routines and habits can be shaped by objects, durable fittings and structures (referred to as ‘props’, ‘infrastructure’ and ‘stage’). When settings are ‘disrupted’ and ‘reconfigured’, objectives for desirable habitual behaviour can be created within the setting through new combinations of props, infrastructure, and stage reinforced by ‘competencies’, ‘roles’, ‘routines’ and ‘norms.’ Examples of poultry-keeping behaviour setting components are provided in Table 2.

**Table 2:** Definitions and examples of settings components.

Dimension	Definition	Examples from poultry settings
Stage	The area of the compound in which the activity takes place e.g. a bathroom.	Birdkeeping ‘stage’ comprising chicken housing/coop, run, and range (where free ranging occurs).
Infrastructure	The durable physical elements of the setting that were employed to complete relevant behaviours e.g. taps, basins.	Roofing, netting or tarpaulin, fencing, walls and flooring that comprise housing and/or enclose run, coop and range. Concrete paths within and leading to bird-keeping area. Watering and feeding systems if fixed.
Props	The objects manipulated to accomplish setting behaviours e.g. soap or kitchen gadgets in a kitchen setting.	Foot dip, shoes/boots to be used in birdkeeping ‘stage’. Bedding. Feeders and waterers if moveable. Disinfectant. Contact sheets for egg or poultry sales.
Roles	The cooperative strategy employed by an individual concerned with helping to accomplish the setting's objective	Official organisations (Defra/the APHA) and other groups communicating HPAI-related risks, regulations and recommendations to minimise exposure and transmission.
Competencies	The embodied and cognitive skills required to accomplish an individual's role in the setting.	Poultry keepers' knowledge of the HPAI-related regulations plus ability and willingness to comply with them.

Given the transferability of IBM-WASH and settings theory to broader public/environmental health-focused interventions requiring multi-level approaches [48], we selected them as frameworks for identifying how different contextual and psychosocial factors influenced compliance with HPAI prevention measures among UK-based smallscale poultry keepers. As the IBM-WASH ‘technology’ dimension was less relevant to our study's focus on compliance with regulatory measures, we focused the third dimension on recommendations developed in collaboration (‘co-produced’) with smallscale poultry keepers. The value of doing so lies in the potential of such recommendations to be understood, and easily acted on by smallscale poultry keepers and, in turn, to promote more effective and sustained biosecurity improvements that reduce the risk of HPAI transmission to humans as well as birds.

## 3 Methodology

This interdisciplinary study presents qualitative insights from directly relevant open-ended questions in a larger online survey (Supplementary file 1) disseminated to smallscale poultry keepers (*n* = 1,559 responses) during the 2021–22 HPAI season. This information is coupled with data from a participatory workshop (*n* = 21 participants) with a subset of the survey respondents (Table 3). Further details on the methodological approach are provided in Supplementary file 2. Anonymised quotes from survey and workshop participants that appear in Section 4 are reproduced verbatim. They are denoted by survey respondent number (e.g. Q123) or by workshop group discussion number (W1–3) as workshop participants were divided into three facilitated groups. Ethical clearance was granted by Nottingham University's School of Veterinary Medicine and Science Committee for Animal Research and Ethics.

**Table 3:** Workshop participant details.

Participant number	Number of birds owned	Types of bird owned	How easy they found housing measures to comply with	Place of residence	Region
01	11–20	Chickens	Very easy	Nottingham	East Midlands
02	21–50	Chickens, Ducks	Easy	Kingston upon Thames	Greater London
03	21–50	Chickens, Ducks, Game Birds, Peafowl	Very difficult	Stoke-on-Trent	West Midlands
04	200+	Chickens	Very difficult	York	North East
05	1–10	Chickens	Okay	Nottingham	East Midlands
06	1–10	Chickens	Okay	Sheffield	East Midlands
07	1–10	Chickens	Easy	Worcester	West Midlands
08	1–10	Chickens	Very easy	Derby	East Midlands
09	11–20	Chickens, Ducks	Okay	Llandrindod	Wales
10	1–10	Chickens	Very difficult	Coventry	West Midlands
11	1–10	Chickens	Impossible	Northampton	East Midlands
12	1–10	Chickens	Okay	Edinburgh	Scotland
13	1–10	Chickens	Easy	Sutton	Greater London
14	200+	Chickens, Geese, Quail	Very difficult	Stockport	North West
15	1–10	Chickens	Okay	Nottingham	East Midlands
16	11–20	Chickens	Very easy	Oxford	South East
17	21–50	Chickens	Okay	Peterborough	East
18	1–10	Chickens	Difficult	Derby	East Midlands
19	1–10	Chickens	Difficult	Derby	East Midlands
20	21–50	Chickens, Ducks, Geese	Okay	Bridgwater	South West
21	1–10	Chickens	Very difficult	Southwell	East Midlands

### 3.1 Questionnaire survey

**Fig. 1:**
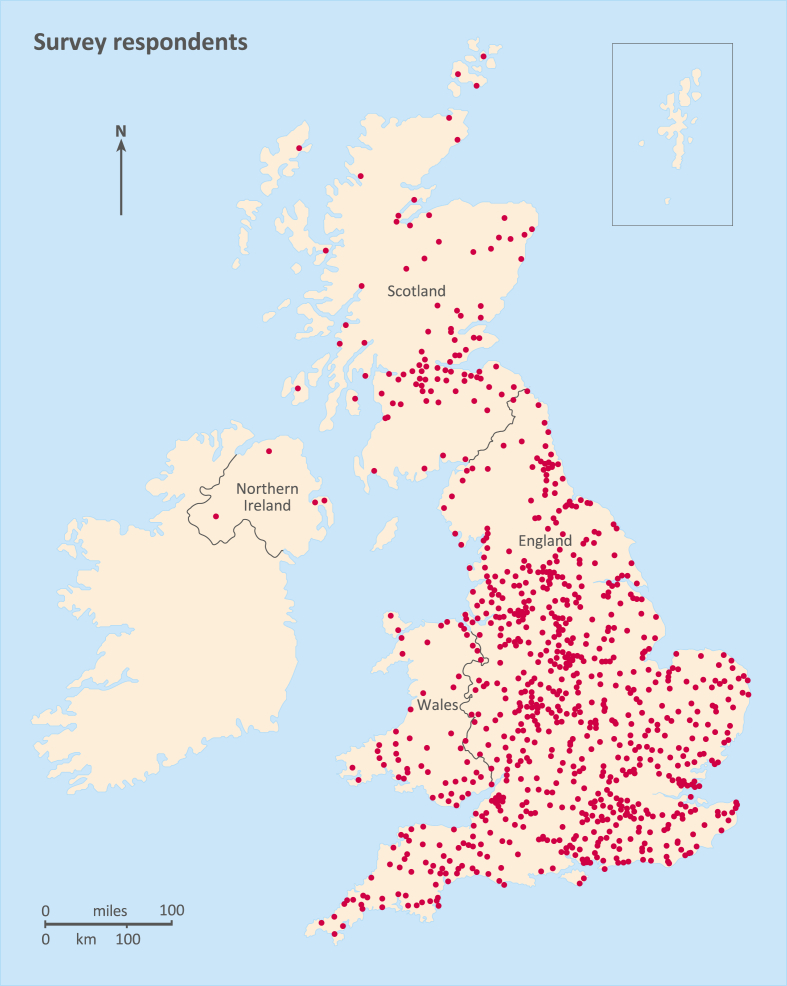
Distribution of respondents to HPAI online survey for smallscale poultry keepers, December 2021–March 2022.

The online questionnaire survey sought to gather data on smallscale UK-based poultry-keeping practices that included how understandings and perceptions of HPAI regulations were disseminated via chicken-keeping websites (e.g. British Hen Welfare Trust) and poultry themed Facebook groups (including ex-commercial hen re-homing organisations). Since there is no agreed definition of the term ‘backyard’ poultry keeper [12], we targeted participants who self-identified as ‘smallscale’ poultry keepers. The majority kept fewer than 50 birds so were not legally required to register their flocks. The survey was open from December 14, 2021 to March 31, 2022 and elicited responses from across the UK (Fig. 1). For the purposes of this paper, open text responses to questions 14 and 16 were analysed qualitatively to explore understandings of AIPZ regulations and elucidate barriers to compliance. Information generated from closed questions 11 through 13 (awareness of the recent outbreak and sources of information), 15 (ease of compliance with housing measures) and 20 (willingness to pay for vaccination) is used for context. A complete analysis of quantitative data from the survey is provided in McClaughlin et al. [51].

### 3.2 Participatory workshop

A total of 637 survey respondents indicated a desire to receive a copy of the survey outcomes. A summary of these was provided via email and recipients were invited to a workshop in July 2022. All participants who accepted the invitation were accommodated at the workshop. Prior to attending, participants were invited to send photographs of their poultry keeping settings to aid discussion during the workshop.

Building on South-North knowledge transfers implicit in the use of IBM-WASH and ‘settings’ theory (both designed for low-income contexts) in the conceptual analysis, the workshop drew inspiration from qualitative research in low- and middle-income countries on backyard poultry keepers' perceptions and responses to HPAI-related risks [[32], [33], [34], [35], [36], [37], [38],^40^,^41^]. Assisted by facilitators, the three workshop discussion groups (W1–3) simultaneously explored smallscale poultry keepers' HPAI-related information sources, their levels of trust in these sources, perceptions of HPAI-related risk and understandings, perceptions and responses to government regulations and communications regarding biosecurity and housing measures. Participants were encouraged to identify aspects of the regulations that they found unclear or were unable (or unwilling) to comply with.

Participants’ photographs of their poultry pens and housing were made available in the workshop to aid discussions of how they interpreted AIPZ regulations and to generate practical suggestions for reconfiguring poultry setting props and infrastructure in cheap and effective ways to improve long-term biosecurity. Lastly, drawing on community-focused studies of how COVID-19-related public health communications were understood and acted on [52], the facilitators and participants discussed how HPAI-related messaging and behaviour change technologies could be targeted more effectively at smallscale poultry keepers.

The resulting ‘co-produced’ suggestions and recommendations are outlined in section 4.3 and key findings from the study have been communicated to stakeholders including the APHA, Defra, online poultry-keeping groups, ex-commercial hen rehoming groups, the British Poultry Veterinary Association (BVPA) and the Poultry Health and Welfare Group (PHWG). Presentations to the BVPA and the PHWG reached poultry veterinary surgeons working in private poultry practice, academia, industry and government as well as members of the British Poultry Council, the British Egg Industry Council, the National Farmers Union and the Game Farmers Association.

### 3.3 Data analysis

Workshop discussions were recorded, transcribed and thematically analysed alongside visual and written information produced by participants (e.g. comments on the HPAI regulations, drawings and photographs). Manual inductive coding was undertaken on these data and also on open text responses to survey questions 14 and 16 to identify key themes. Subsequently, manual deductive coding utilised the IBM-WASH framework to identify barriers to compliance with HPAI-related regulations across the three dimensions and five levels. Summary statistics for the closed survey questions were generated in Microsoft Excel (v2303).

## 4 Results

Table 4 summarises the key findings from the questionnaire survey and workshop. The findings are themed according to the IBM-WASH model structure (Table 1) with co-produced recommendations replacing the ‘technology’ dimension. We consider these findings in more detail below, focusing on each level of the three dimensions (contextual, psychosocial, and co-produced recommendations) in sequence.

**Table 4:** Summary of barriers to compliance with HPAI housing measures and co-produced recommendations.

Level^1^	Contextual factors	Psychosocial factors	Co-produced recommendations/suggestions
Societal/Structural	HPAI-related regulations unclear and poorly targeted at smallscale poultry keepers. Climatic and geographical factors hinder compliance if housing is destroyed.	Poor advocacy and HPAI-related communications to poultry keepers and general public. Avian influenza prevention zone regulations unclear and confusing. Failure by Defra/the APHA to connect with smallscale keepers; especially those who see their birds as pets.	Clear, simple targeted messaging for smallscale keepers. Better communication of HPAI-related risk and benefits of adhering to biosecurity and housing measures. Risk assessments reflecting the current UK situation. Strong support for vaccination among smallscale keepers.
Community	Physical environment can hinder reconfiguration of poultry settings and repair following damage.	Shared values, collective efficacy and social integration influenced compliance with housing and biosecurity measures. Stigma associated with non-compliance. Mistrust between different poultry keeping sectors regarding compliance.	Geographically- and time-specific responses that account for varying risk levels. Dissemination of targeted guidance via online poultry communities. Vaccination (if available) as a condition for attending poultry shows.
Household/interpersonal	Available space, household characteristics and competencies influence the ability, labour and time to comply.	Nurture informed descriptive and injunctive norms including outrage at others' non-compliance, fears of being inspected by Defra and shame if found to be non-compliant. Nurture also influenced welfare concerns during flockdown.	Additional communication routes to disseminate HPAI-related guidance to smallscale keepers who don't belong to online or in-person poultry communities (e.g. at vets or feed suppliers).
Individual	Compliance influenced by individuals' time, finances and self-efficacy or competency. Cooperative roles played by friends and livelihood settings also played a role.	Nurture underpinned efforts to balance perceived threat of HPAI with welfare impacts of flockdown. Lack of knowledge of HPAI transmission. Self-efficacy evident in high compliance with housing measures although limited funds or practical skills restricted some keepers.	Guidance on how to comply with housing measures in cheap and achievable ways. Photographs, infographics and videos to illustrate practical solutions.
Habitual	Sustained biosecurity habits challenged by climatic factors, time, finances and a sense of others' non-compliance with housing measures.	Keepers attempted to balance HPAI risk and welfare impacts with backsliding sometimes occurring when long-term housing created health and welfare concerns. Inability to exclude small birds from poultry areas.	HPAI messaging, guidance, illustrations of compliant housing and cheap suggestions for achieving it to be disseminated outside flockdown and in places likely to reach less internet-savvy keepers (e.g. veterinary practices, feed bags). Promote vaccination as an alternative to housing measures.

*Notes*:^1^ See Table 1 for level descriptors associated with the contextual and psychosocial factors. The descriptor for habitual level psychosocial factors has been adapted here for compliance with biosecurity and housing measures.

### 4.1 Contextual influences

Alongside seasonal and geographical factors that influence the risk of HPAI introductions into poultry (e.g. proximity to the breeding and wintering sites of migratory waterbirds and the abundance and habitat use of such birds; see Hill and colleagues [53]), the contextual dimension highlights the need to communicate HPAI-related policies and regulations more effectively to smallscale poultry keepers.

#### 4.1.1 Societal/structural

Answers to survey questions 11 and 13 indicated that over 99% of the survey respondents were aware of the UK-wide 2021–22 mandatory housing order (‘flockdown’) with 71.5% mentioning official sources (Defra or the APHA) and 27% obtaining information from the British Hen Welfare Trust (BHWT). Responses to question 14 and workshop discussions suggested that key messages about keeping poultry in covered or netted runs and away from wild birds were well understood but pointed to limited awareness of the wider biosecurity measures outlined in Schedule 1 of the AIPZ regulations [54]. A few survey respondents (4%) reported confusion over these regulations while others expressed frustration about their lack of clarity and their inability (or unwillingness) to comply:

*Jump through a ridiculous amount of hoops which are expensive and often impossible to comply with (Q163).*

These themes were elaborated on by workshop participants who expressed concern that the regulations were confusing, targeted at commercial poultry establishments and not necessarily applicable to smallscale keepers:

*Thought these were only for commercial set ups (W3).*

Regulations on the recording of vehicles entering the premises, and the movement or sale of poultry and eggs, were viewed as overly bureaucratic and unachievable:

*How you keep track of where the eggs are going if you are using an honesty box to sell eggs? (W2).*

Also poorly understood were the ‘welfare grounds’ that permitted birds to be kept in “fully enclosed or netted outdoor areas” [9] rather than being ‘housed’ and the types of cover allowed in such areas. Some workshop participants also expressed confusion about the term ‘biosecurity’ and the use of disinfection measures like foot dips:

*Not sure what biosecurity means (W3).*

*Confused how to do footdips (W3).*

Climate and geography are important societal/structural factors that influence the scale and distribution of HPAI outbreaks along with the effectiveness of housing measures and smallscale keepers' ability to comply with them. In previous years, UK-based HPAI outbreaks have demonstrated a distinct seasonal peak between October and March, falling thereafter with higher spring and summer temperatures [55]. Climatic and geographical factors also influence keepers’ abilities to maintain effective biosecurity as poultry settings are more likely to be damaged by strong winds, storms, and snowfall in exposed areas:

*Storm Arwen destroyed my hard work !! (Q132).*

#### 4.1.2 Contextual – community

Such difficulties are also apparent at the community level where the physical environment and access to resources can hinder efforts to reconfigure poultry-keeping infrastructure to provide secure housing during flockdown. Some keepers mentioned taking extreme measures to comply:

*Don’t have enough housing or runs that can stand up to Orkney winds to house all the poultry, so forced to cull some that should have been able to live long healthy lives (Q17).*

Others reported that natural characteristics hindered the reconfiguration of poultry-keeping ‘stages’ and infrastructure to comply with housing measures:

*We just can’t get the overhead cover fully protective around the trees. Even though we’ve reduced the area in use it’s still really difficult and is not 100% effective (Q558).*

#### 4.1.3 Contextual – interpersonal

Table 4 highlights how, at the household and interpersonal levels, respondents' abilities to comply with housing and biosecurity measures often reflected the nature of existing poultry-keeping ‘stages’ and the competencies, labour, time and space available to reconfigure them. Common themes included a “lack of advance warning” (W1) of flockdown, an inability to cover existing runs or to fundamentally change the nature of birdkeeping infrastructure:

*Not enough enclosed space and not able to extend it (Q499).*

*Can’t implement the disinfection measures as the coop is dirt floored (Q1).*

Waterfowl keepers, in particular, reported welfare issues that made it difficult to implement housing measures:

*I don’t have enough indoor space to keep my ducks and their water. Ducks have to be able to put their heads in water to be able to eat properly. When indoor spaces are wet, mould spores kill ducks. I have tried to net an area but it’s not successful and expensive (Q600).*

Some respondents also mentioned specific household circumstances and roles that affected their ability (competency) to reconfigure poultry-keeping infrastructure to comply with housing measures while others managed to obtain help from friends with ‘do-it-yourself’ (DIY) tasks:

*I’m currently pregnant, partner abandonment and my DIY guy has broken his hand so really struggling to get the work done (Q598).*

*We purchased a larger 3m × 3m walk in run as the netting run was no longer legal … As my husband is disabled I needed a friend to help me erect it. It took a day and a half to two days to erect safely and to put a tarpaulin cover over the top to act as a roof (Q765).*

#### 4.1.4 Contextual – individual

At the individual level, a key barrier to compliance was the cost of implementing housing measures (Table 4) with some spending significant sums of money to keep their birds housed:

*I managed by spending several thousand quid but if I didn’t have the money I wouldn’t have been able to house them adequately without causing animal cruelty (Q1233).*

The intersection of poultry-keeping and livelihood settings also presented difficulties for some respondents, with one reporting backsliding when the polytunnel they housed their chickens in was required for other purposes (Q1268). Another found biosecurity and housing measures difficult to implement within their working environment:

*We cannot keep foot dips spotless clean, we are a working farm with muddy fields. Shutting geese in together when it is nearing breeding season has caused immense fighting, netting pens during a storm is ridiculous. These rules are ok for a commercial setting or those with a couple of back garden hens but shutting in all my breeding stock has made it impossible to keep them clean despite spending all hours trying to sort stuff (Q166).*

#### 4.1.5 Contextual – habitual

**Fig. 2:**
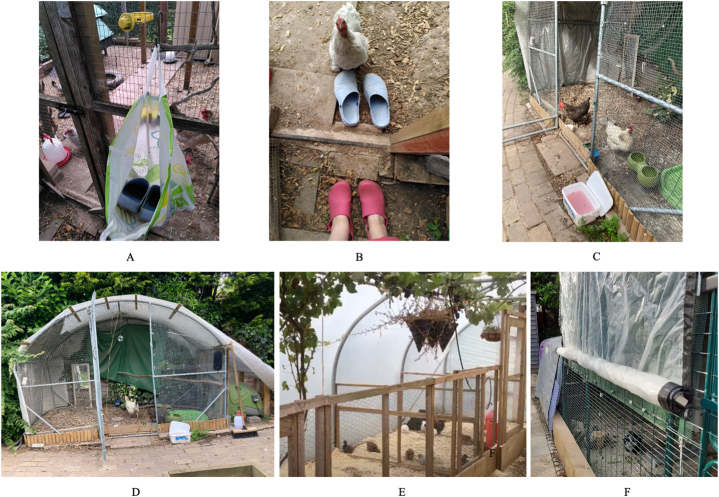
Illustrations of innovative reconfigurations of poultry-keeping props and infrastructure by workshop participants. (A) Garden clogs stored at the entrance to a chicken coop. (B) Garden clogs positioned for use at the coop entrance. (C) A foot bath positioned at the entrance to a coop. (D) A covered chicken coop with a foot bath outside and ‘enrichment’ features (mirror, dust bath, roosting bars, log segment) inside to reduce boredom during ‘flockdown’. (E) A polytunnel being used to house chickens during ‘flockdown’. (F) A coop featuring side covers that can be rolled down in rainy weather or raised on warm or windy days.

The majority of survey respondents reported compliance with AIPZ regulations while they were in force, with only 10% noting an inability to implement housing measures for a range of practical, financial, or welfare-related reasons. Whilst disparities between self-reported compliance with health-related measures and actual behaviours are not uncommon [52,56], photographs shared by workshop participants illustrated the significant efforts that had been made to house birds in high welfare settings; see Fig. 2. Innovative reconfigurations of poultry-keeping props and infrastructure designed to facilitate the routine and sustained implementation of biosecurity measures included hanging a bag containing spare shoes or boot covers at the poultry run entrance to prompt a change of footwear (Fig. 2A and B). Other keepers placed foot dips and cleaning equipment at the entrance to prompt their use (Fig. 2C and D). Some photographs demonstrated keepers’ advanced DIY competencies with a polytunnel repurposed as chicken housing (Fig. 2E) and a coop containing retractable side covers that allowed ventilation on warmer days but reduced wind resistance in stormy weather (Fig. 2F). Despite such efforts, significant challenges were noted by both survey and workshop participants (Table 4). The most common reason for backsliding related to inclement weather damaging poultry infrastructure and the practical and financial difficulties in making repairs:

*We did implement, but it was destroyed by the weather and we can’t afford to rebuild (Q269).*

Backsliding was also linked to non-compliance among other poultry keepers, especially towards the end of the housing measures. One workshop participant recalled conversations with a local postman about many “birds out on farms locally” (W2), while another observed “low compliance regarding flockdown” (W2).

### 4.2 Psychosocial

As illustrated in Table 4, the psychosocial dimension captures interesting issues around respondents' trust of the organisations responsible for HPAI-related regulations and communications (primarily the APHA/Defra) and their perceptions of risk to their birds’ health from HPAI versus those that they associated with the housing measures.

#### 4.2.1 Societal/context

Although respondents’ awareness of HPAI outbreaks and associated regulatory responses was high, many were critical of how information was communicated (or otherwise) to the general public as well as to smallscale poultry keepers:

*Big delay in notifiying (sic) people [in] 10km zone (general public) 6–8 weeks after outbreak where I live. How will this help? (W1).*

Some workshop participants described AIPZ regulations as ‘hard to access’ or ‘unclear’ and struggled to establish whether and how they needed to reconfigure their poultry-keeping settings and routines:

*Regulations and advisory guidance from APHA assumes you have agricultural training and understand all the terminology (W3).*

Others expressed frustration that the APHA/Defra failed to communicate why poultry keepers should abide by the regulations:

*No communication on why this [AI] is ‘bad’. Only that chickens might die. Not thinking about human health (W1).*

Echoing Sutherland and colleagues’ findings [57], workshop participants highlighted a distrust for the APHA/Defra on issues relating to animal welfare with reference to examples of animal culling during the 2001 UK Foot and Mouth Disease outbreak and the decision to euthanise Geronimo the alpaca as part of measures to control bovine TB [[9], [10], [11], [12], [13], [14], [15], [16], [17], [18], [19], [20], [21], [22], [23], [24], [25], [26], [27], [28], [29], [30], [31], [32], [33], [34], [35]] [[36], [37], [38], [39], [40], [41], [42], [43], [44], [45], [46], [47], [48], [49], [50], [51], [52], [53], [54], [55], [56], [57], [58], [59]]:

*People have lost confidence in DEFRA (W2).*

The perceived failure of the APHA/Defra to connect with smallscale poultry keepers and appreciate that many see their birds as “pets not commodities” (Q490) was an important theme.

#### 4.2.2 Psychosocial – community

At the community level, shared values, collective efficacy, and social integration influenced respondents’ attitudes towards, and compliance with, housing and biosecurity measures (Table 4). Many of the poultry groups that advertised our survey routinely deleted posts indicating non-compliance with flockdown, criticised keepers who refused to house their birds and provided (often unsolicited) advice on reconfiguring poultry-keeping infrastructure, props and routines to improve biosecurity. To avoid stigma associated with apparent non-compliance, those who posted on such sites often contextualised photographs of poultry or housing with explanations that they were taken before flockdown. Echoing this, one workshop attendee who had struggled to adhere to the 2021–22 housing measures described their participation as “a bit like a chicken speed awareness course” (W2). This sentiment was echoed in their concerns about being reported for breaking flockdown rules:

*Occasionally our chickens would ‘escape’ onto the country lane outside our house and I was worried I would get ‘shopped’ (W2).*

Workshop discussions also highlighted a sense of mistrust and institutional stigmatisation associated with commercial stakeholders that blamed smallscale poultry keepers for the onwards transmission of HPAI:

*Smallscale poultry keepers are demonized by big poultry producers, lobby groups Defra, NFU etc. who find it convenient to blame small keepers for AI suggesting we don’t keep our chickens properly which doesn’t help to raise the profile of the disease or encourage compliance (W2).*

Meanwhile, smallscale keepers who tried hard to comply with AIPZ regulations expressed frustration about non-compliance in the commercial sector, especially when they felt that they and their birds were making sacrifices:

*Ach, it’s all bollocks, really. Last time, I rang the DEFRA, and they said my set up of fencing them in was ok, so that’s what I’ve done this time, with some netting on the top to stop the buggers getting out. It’s better this time, because at least the big farms appear to being doing something. Last time, the poultry farms just ignored it, while my hens were all sad … It makes me cross my pet hens have to suffer to protect industrial, cruel farming (Q950).*

#### 4.2.3 Psychosocial – interpersonal and household

Many themes identified in the community section were also evident at interpersonal and individual scales. ‘Nurture’-related concerns (i.e. concerns over bird welfare – especially housing measure-related restrictions – and the need to protect them from exposure to HPAI) were frequently articulated by participants while a perceived lack of compliance by other keepers often elicited disapproval for breaching ‘descriptive and injunctive norms’ [45] associated with good poultry keeping (Table 4). One workshop participant, outraged at the risk presented to their birds by others' lack of compliance, wrote:

*Whilst dog walking! Saw several farms that had taken no measures. DEFRA phoned but not interested (W1).*

Some respondents expressed fears of being inspected by Defra and the shame they would feel if found to be non-compliant. Echoing this, photographs that workshop respondents provided of their poultry setups clearly illustrated aspirations to depict a combination of good biosecurity and bird welfare (Fig. 2A–F).

Discussions of how respondents reconfigured their birdkeeping ‘stages’ during flockdown prompted numerous nurture-related conversations that were echoed in the questionnaire responses. Poultry breeders mentioned difficulties associated with male birds fighting when confined:

*Can only comply with housing measures by keeping them [male birds] in breeder cages. Might as well put them in battery cages (W2).*

Waterfowl keepers also reported welfare-related concerns that sometimes made them unwilling to house their birds:

*Our geese are still outside as it’d be cruel to confine them that much (Q686).*

*Not possible to house my ducks under cover, humanely (Q701).*

Similar concerns were reported among chicken owners who refused to house their birds “because it's cruel” (Q606) or who complied but attributed chicken deaths and ill health to restricted flockdown settings:

*Complying as best we can, but … Feel awful trapping my girls in a large run, when they have over half an acre … My chickens are pets, not a commodity … Annoying when many in the area don’t bother to comply … Had lots of illness with my girls after previous lockdown, due to being locked in, which made me very cross (Q490).*

*Too small a house–direct cruelty to my chickens–last year they were VERY DISTRESSED BY IT, they killed the bottom of the pecking order hen (Q930).*

Some used highly emotive language such as “imprisoned” (Q17) and “jail” (Q146) to express distress about confining their birds:

*Those imprisoned are less healthy and happy than if they were in their usual extremely high welfare extensive free ranging, including in woodland areas … It is appalling that we have to take measures designed for intensive factory farming of poultry & that we’ve had to kill perfectly healthy birds we just couldn’t get into the required runs (Q17).*

#### 4.2.4 Psychosocial – individual

As Table 4 and Fig. 2 show, themes of nurture and perceived threat were very apparent at the individual level. A few keepers, who regarded their birds as pets, brought them into their homes as an HPAI preventive measure:

*In conservatory until end bird flu season (Q830).*

*Duck has his own ‘duck hotel’ to sleep in indoors with us and moves around freely indoors during the day (lots of plastic sheeting down!) (Q632).*

Many others discussed efforts to balance their perceptions of HPAI-related threats with concerns about the welfare-related impacts of housing measures:

*… I will not keep them housed only in their chicken coop, despite it being oversized for the number of chickens I have. Chicken welfare needs to be balanced against the risk (Q985).*

Responses like these tended to reflect resentment about official interference in what were considered personal issues coupled with broader mistrust for Defra:

*… no birds here are falling out of the sky, no one else is bothering and as usual defra over reacting and restricting them causes more problems … (Q775).*

Echoing this, workshop discussions indicated that smallscale keepers often lacked knowledge about HPAI-related transmission or prevention. Some respondents criticised the APHA/Defra for their poor credibility and failure to emphasise the importance of housing and biosecurity measures:

*Emphasis on “no risk to public, no risk to food supply” doesn’t encourage compliance (W2).*

*Information would not be as readily accessible for those who don’t trust Defra (W3).*

In relation to self-efficacy, 42% of survey respondents reported finding housing measures easy or very easy to comply with, while 39% found them ‘OK’, 16% found them ‘very difficult’, and 3% ticked the ‘impossible’ option for Question 15. Question 16 which invited explanations from those unable to implement the housing measures was completed by 10% of respondents. The most common were practical or competency-related issues (103 responses), welfare concerns (42 responses) and financial constraints (15 responses) with some respondents mentioning all three. Several mentioned an inability to net or roof poultry runs:

*I haven’t been able to entirely cover my run. Too difficult a diy job for me to manage (Q268).*

Others indicated that they had the skills and resources to adapt their infrastructure to comply with housing measures but hesitated because of welfare-related concerns:

*Can implement, fairly easily, but concerned about the welfare of my birds … totally unnatural and stressful environment causing starvation and death. Rescue hens; having tasted complete freedom, they hate being in lockdown again. Very stressed (Q1230).*

#### 4.2.5 Psychosocial – habitual

At the habitual level, partial compliance and backsliding reflected keepers’ efforts to reconcile nurture-related concerns about minimising HPAI-related risk with the welfare impacts of long-term housing measures:

*I can’t leave the ducks without a bath, so they have half an hour a day in a stableyard to have a bath (Q113).*

Failure to exclude small birds from poultry runs or contain housed birds were also mentioned as barriers to sustained compliance with biosecurity measures:

*It doesn’t matter what I do sparrows still manage to get in also my chickens are escape artists! (Q1224).*

Backsliding was also mentioned by respondents who abandoned housing measures for welfare reasons and reconfigured other aspects of their poultry-keeping “stages’ to strengthen biosecurity:

*‘Trying to house or pen the geese proved more difficult than trying to land on Mars. 2 days the geese were shut in, the only thing they ate in those 2 days was the damn door frame of the stable. Penned them, they tried to kill each other, separated them – they tried to kill themselves. In the end I’ve massively restricted their grazing and removed their pond. I disinfect everything to minimize the risk but it’s impossible (Q1309).*

### 4.3 Co-produced recommendations

The co-produced recommendations arising from discussions with workshop participants and informed by our survey responses are summarised in the final column of Table 4. Three main areas of action can be identified, namely: (i) the targeting of biosecurity messages more effectively at smallscale keepers; (ii) the provision of more practical guidance on improving and maintaining biosecurity; and (iii) the potential use of vaccination as an alternative control strategy.

#### 4.3.1 Solution – societal structural

At the societal/structural level, a key recommendation was the need for clearer, more targeted biosecurity messaging for smallscale poultry keepers:

*Communications need to be better tailored to different groups (e.g. commercial vs backyard) (W2).*

Participants wanted greater clarity on the meaning of the terms ‘housing’ and ‘inside’, the size/types of netting permitted, and identifying HPAI as the photographs currently on the Defra website were described as “not good” (W1):

*Remove the phrase ‘keep them inside’–that leads to birds in kitchens and conservatories (W3).*

*Information on the size of the netting–something that you can’t push x object through (W2).*

Participants' assessments of existing avian influenza guidance documents produced for smallscale keepers [[60], [61], [62]] indicated that they were far easier to follow than Defra's (2021a) AIPZ regulations [54]. The Welsh Government's document [62] was praised for its clear and simple guidance and the Scottish Government's checklist style biosecurity information for small flock keepers [60] was also well received, although some participants recommended visually more engaging formats:

*Colourful, engaging points/check sheet with bare minimum requirements (W3).*

The APHA's interactive map was considered ‘helpful’ and several participants suggested an app for smallscale keepers:

*An app would be a good place for everyone to get the same info (W3).*

Respondents also felt that HPAI-related risk needed to be better communicated by Defra, the APHA and DAERA as the current messaging fails to outline the benefits and importance of adhering to biosecurity and housing measures:

*Focusing on birds owned by individuals rather than vague appeals to the general good would be more effective (W2).*

One participant recommended that HPAI communications should “take inspiration from the drink driving/seat belt campaigns” (W2), while others emphasized that regulations should be underpinned by robust risk assessments that reflect the current HPAI situation:

*Risk assessments that keep up with the changing nature of the disease (W2).*

Vaccination was another topic relevant to the societal/structural level with 93% of survey respondents indicating a willingness to pay for a vaccine if one was made available (Question 20). Of these 26% stated that they would pay up to £2.50/dose, 56% would pay £2.50–20/dose and 10% would pay over £20/dose. Workshop respondents also indicated strong support for vaccination with some commenting that it should be mandatory and controlled through bird-keeping licenses. Compliance issues, however, were recognized:

*Make vaccination mandatory–would create buy in but there would be problems with adherence (W2).*

*Introduce a license to keep chickens (this is the case in NI) (W2).*

#### 4.3.2 Solutions – community

Among poultry-showing participants, vaccination offered hope that competitions might resume and there was support for making vaccination a condition of entry:

*Poultry clubs would be behind vaccination initiatives to protect their flock and the ability to go to shows (W2).*

Echoing points about communicating the importance of adhering to HPAI regulations, participants felt that appeals to shared values could encourage uptake if vaccination was introduced:

*Vaccination has wider benefits for puffin or gannet colonies– this could be emphasized to encourage uptake (W2).*

Drawing on Covid-19 analogies, some workshop participants recommended adopting more geographically- and time-specific approaches that account for varying risk levels (Table 4). Suggestions included greater use of zoned approaches (instead of extended UK-wide flockdowns) in areas close to bird migration routes, waterfowl colonies and other high-risk sites (W2) coupled with communications to smallscale keepers about how and why to reconfigure their birdkeeping infrastructure for greater biosecurity:

*Infographics or communication to backyard keepers [e.g.] if you live near an estuary … if you live near a wildlife reserve … consider having a solid roof on your run (W2).*

For the effective dissemination of HPAI-related information, workshop participants recommended disseminating targeted guidance via online poultry communities. Many had used information and advice from such groups when reconfiguring their birdkeeping infrastructure and props to improve biosecurity and suggested that they could assist with future flockdown preparations with information on such matters as:

*where to buy netting [that complies with biosecurity guidance] so it is easy for people to get it (W2).*

*A list of acceptable and easy to access foot dip disinfectants geared to small-scale keepers (e.g. Dettol, bleach) (W2).*

#### 4.3.3 Solutions – household/interpersonal

To reach smallscale keepers who do not belong to online or in-person poultry communities or “those who don't trust Defra” (W3) workshop respondents recommended additional HPAI-related communication routes:

*Ensure … farm or poultry suppliers are disseminating information about restriction zones (W3).*

*Vet practices could be used more (to disseminate information) (W2).*

The importance of vet practices was also highlighted in discussions about vaccination as a possible future policy.

#### 4.3.4 Solutions – individual

At the individual level, knowledge, competencies, self-efficacy, and cost were important themes relating to compliance with HPAI measures and attitudes towards vaccination. Workshop participants suggested that keepers who regarded their birds as pets were more likely to vaccinate although they warned that cost would influence uptake or compliance if vaccination became mandatory:

*Not all would spend as much to vaccinate chickens as they would for dogs and cats (W2).*

Guidance on how to comply with housing measures in cheap and achievable ways was viewed as particularly valuable for improving biosecurity in smallscale poultry settings:

*Requirements for backyard keepers must be simple, practical and cost effective (W2).*

Workshop participants therefore recommended targeting simple messaging at smallscale keepers coupled with photographs and videos to illustrate practical solutions:

*Bite-size videos – tiktok (W3).*

Examples of good practice included a video by the Surrey Poultry Vet [63] demonstrating the placement of garden clogs outside poultry pens to facilitate a change of footwear on entry.

#### 4.3.5 Solutions – habitual

This type of disruption to poultry-keeping ‘stages’ was acknowledged as easy to implement with potential to promote sustained use of biosecurity measures as placing ‘props’ such as garden clogs or foot baths prominently reminds keepers to habitually use them (Fig. 2A–D). Recalling the rapid implementation of the 2021–22 flockdown, workshop participants highlighted the benefits of using the summer to reconfigure birdkeeping infrastructure with a view to promoting long-term biosecurity and enabling easy conversion to housing measures when required. Suggestions for doing this cheaply included searching freecycle sites for timber, fence panels, netting, chicken wire and tarpaulin to create, repair or enlarge poultry housing and cover outdoor runs. Survey respondents also reported using children's playhouses, greenhouses, polytunnels, gazebos, and even old caravans as additional poultry housing. To facilitate such preparations, workshop participants recommended disseminating illustrations of compliant housing and cheap, practical suggestions for achieving it with information placed in vet practices, feed stores, and on poultry feed bags to help reach less internet-savvy keepers. One participant suggested that poultry housing manufacturers could offer summertime discounts on biosecurity-enhancing items (covered runs, foot dips, netting, disinfectant, rodent proof feeders etc.) to encourage preparedness. Regarding the sustainability of a vaccination program, if implemented, respondents felt that uptake would increase if this removed the need to house birds during HPAI outbreaks.

## 5 Discussion

In high-income as well as in low-income countries, a lack of information on the number of smallscale poultry keepers and their knowledge of and willingness to comply with recommended biosecurity practices hinders the implementation of measures to control HPAI [14,18,19,21,22,[32], [33], [34], [35], [36], [37], [38], [39]]. The aim of this study was to explore the understandings and responses of smallscale poultry keepers in the UK to governmental directives to control HPAI exposure and onwards transmission. Particular emphasis was placed on how respondents perceived HPAI-related risks, their willingness and ability to comply with compulsory HPAI prevention measures and barriers to improving (and maintaining) biosecurity in their poultry keeping settings.

Since our online questionnaire survey was disseminated primarily via social media, the results may over-represent the experiences of poultry keepers with good internet access, familiarity with social media, interest in avian influenza or confidence in their poultry-related knowledge [14]. Engagement with the survey was particularly high among members of ex-commercial poultry rehoming groups (e.g. British Hen Welfare Trust and Fresh Start for Hens), introducing potential bias from the over-representation of such respondents (many of whom view their birds as pets). In combination with the tendency for online poultry groups to discuss HPAI-related housing measures and biosecurity recommendations, this may overestimate levels of compliance and knowledge among the wider poultry-keeping community. There was also potential for bias resulting from the self-selection of workshop participants, as attendees at such events may be more likely to consider themselves knowledgeable about, or have particular interests in, the topics being discussed. The decision to hold an in-person workshop and its location in the UK Midlands may also have limited the pool of potential attendees.

Ideally, a comparison of participants’ geographical location, age group and gender would be performed against the organisation membership that they formed part of. However, a lack of demographic information on participants, coupled with legal matters relating to data protection in the United Kingdom (UK-GDPR) and associated difficulties of accessing information on members of the target organisations, has precluded a systematic analysis of selection bias in the current study.

In contrast to some other studies in high-income contexts [18,21], respondents' awareness of HPAI was very high and key messages associated with the housing measures were well understood although this may reflect the survey's completion during a period of mandatory housing measures [14]. Echoing findings from the UK [14,64], the US [21], Canada [65,66] and New Zealand [22,67] respondents' knowledge of biosecurity requirements and why these are put in place was less comprehensive, with some expressing scepticism or confusion about the potential for onwards transmission of HPAI to humans and other animals. Observations of poor knowledge about the risk of contracting other diseases (e.g. Salmonella) from backyard poultry [14,18,19,23,64] were also evident in our study, as were the dangers of poorly-targeted HPAI-related messaging. By way of example, some survey participants ‘housed’ poultry in their homes as they misunderstood (or alternatively made extreme efforts to comply with) housing measures. The capacity or willingness of other respondents to implement mandatory housing measures reflected behavioural determinants similar to those identified by Sultana and colleagues [34] in Bangladesh with context-specific geographical factors plus a lack of funds, time, space or practical skills often hindering sustained compliance.

IBM-WASH [45] provided a valuable framework for exploring these barriers to adherence and making recommendations for addressing them while settings theory [49] provided useful insights into how poultry-keeping settings and routines could be cheaply and conveniently reconfigured to improve long-term biosecurity and reduce HPAI exposure and transmission risk. The contextual dimension highlighted challenges to the uptake and sustained implementation of biosecurity and housing measures at all five levels, with climatic and geographical factors often causing backsliding. It also revealed a strong desire by most smallscale poultry keepers to simultaneously protect their flocks and comply with regulations. Many viewed their birds as pets and expended significant amounts of time and money in creating predator-proof, biosecure and high welfare settings for them (Fig. 2A–F). The language such keepers used when discussing their birds contrasted sharply with official communications about HPAI and while the current regulations may be appropriate for, and comprehensible to, commercial poultry farmers, workshop participants favoured a more tailored approach for smallscale keepers.

Illustrating the value of a multi-level framework, the psychosocial dimension identified overlaps between community and interpersonal levels in relation to shared values, collective efficacy, social integration, stigma, injunctive/descriptive norms, shame, aspirations, and nurture. Some of these overlaps reflected respondents’ engagement with online poultry groups. While some keepers on these sites were criticised (or their posts removed) if they indicated non-compliance with housing measures, members could also learn how to reconfigure their birdkeeping infrastructure and props to comply with biosecurity and housing measures (e.g. Sections 4, 4.2.2.3.2). Exploring the potential of settings theory to inform future HPAI-prevention initiatives in different contexts would be a valuable area for future research.

Co-produced recommendations derived from our survey responses and workshop discussions focused on three main issues. First was the need for improved communications about HPAI-related risk and biosecurity measures. Respondents recommended the dissemination of clearer, simpler and more targeted messages to smallscale keepers with measures taken to reach those with limited internet access/use, as well as online communities. More sensitive approaches were advised for communicating with keepers of pet poultry, especially regarding emotive topics like culling. A second set of recommendations focused on providing visual guidance on cheap and easily achievable ways to reconfigure smallscale poultry-keeping settings to comply with housing measures and to disseminate this during the summer when good weather would facilitate preparations for future flockdowns. The third recommendation focused on vaccination (if permitted) as a potentially popular HPAI-related risk management strategy among smallscale poultry keepers; especially if it removed the need for housing measures.

## 6 Conclusions

Findings from the study have the potential to shape future responses to HPAI outbreaks in the UK and beyond; notably by indicating how policy-makers and key stakeholders can communicate more effectively with smallscale keepers, highlighting their views on vaccination and identifying key barriers to compliance with mandatory housing orders. The co-produced recommendations have been communicated to key stakeholders, including the APHA/Defra. It is our hope that these recommendations will help to promote sustained biosecurity improvements among smallscale keepers, inform efforts to control HPAI outbreaks and reduce disease transmission pathways from backyard flocks to wild birds, humans and other animals in a range of different contexts.

## Ethics statement

This study was reviewed and approved by the School of Veterinary Medicine and Science Committee for Animal Research and Ethics at the University of Nottingham. The approval number is 3550220208. All participants provided informed consent to participate in the study. All participants provided informed consent for the publication of anonymised quotes and workshop attendees provided informed consent for the publication of images, visual and written information produced or supplied during and in advance of the workshop.

## Author contribution statement

Sarah Jewitt, Matthew Smallman-Raynor, Emma McClaughlin: Conceived and designed the experiments; Performed the experiments; Analyzed and interpreted the data; Wrote the paper.

Michael Clark, Stephen Dunham, Rachael Tarlinton: Conceived and designed the experiments; Performed the experiments.

Sol Elliott: Conceived and designed the experiments; Performed the experiments; Analyzed and interpreted the data.

Alastair Munro, Tamsin Parnell: Performed the experiments; Analyzed and interpreted the data.

## Data availability statement

Data will be made available on request.

## Declaration of competing interest

The authors declare that they have no known competing financial interests or personal relationships that could have appeared to influence the work reported in this paper.

## References

[1] Smallman-Raynor M.R., Cliff A.D., A D (2008). The geographical spread of avian influenza A(H5N1): panzootic transmission (December 2003–May 2006), pandemic potential, and implications. Ann. Assoc. Am. Geogr..

[2] Stegeman A. (2004). Avian influenza A virus (H7N7) epidemic in The Netherlands in 2003. J. Infect. Dis..

[3] Gilbert M. (2006). Anatidae migration in the western Palearctic and spread of highly pathogenic avian influenza H5NI virus. Emerg. Infect. Dis..

[4] Terregino C. (2007). Active surveillance for avian influenza viruses in wild birds and backyard flocks in Northern Italy during 2004 to 2006. Avian Pathol..

[5] Bavinck V. (2009). The role of backyard poultry flocks in the epidemic of highly pathogenic avian influenza virus (H7N7) in The Netherlands in 2003. Prev. Vet. Med..

[6] Freath L. (2022). https://assets.publishing.service.gov.uk/government/uploads/system/uploads/attachment_data/file/1060076/hpai-europe-220307.pdf.

[7] UK Government (2023). Bird Flu (Avian Influenza): Latest Situation in England.

[8] Jarvis L. (2022). https://www.nationalgeographic.co.uk/environment-and-conservation/2022/09/the-uks-largest-avian-flu-outbreak-has-left-millions-of-birds-dead-and-scientists-extremely-concerned.

[9] Defra (2021). https://www.gov.uk/guidance/avian-influenza-bird-flu.

[10] Defra (2019). Notifiable Avian Disease Control Strategy for Great Britain.

[11] (2023). Defra. Mitigation Strategy for Avian Influenza in Wild Birds in England and Wales.

[12] Smith G., Dunipace S. (2011). How backyard poultry flocks influence the effort required to curtail avian influenza epidemics in commercial poultry flocks. Epidemics.

[13] Souvestre M. (2019). Role of backyard flocks in transmission dynamics of highly pathogenic avian influenza A(H5N8) clade 2.3.4.4, France, 2016–2017. Emerg. Infect. Dis..

[14] Correia-Gomes C., Sparks N. (2020). Exploring the attitudes of backyard poultry keepers to health and biosecurity. Prev. Vet. Med..

[15] UK Government (2022). Preliminary and Updated Outbreak Assessments for Avian Influenza (Bird Flu) in Europe, Russia and the UK.

[16] APHA (2022). Livestock Demographic Data Group: Poultry population report. Livestock population density maps for GB, using July 2021 data.

[17] MRCVSonline. Chicken (2020). Ownership on the Rise, Survey Reveals.

[18] Elkhoraibi C. (2014). Backyard chickens in the United States: a survey of flock owners. Poultry Sci..

[19] Tobin M.R. (2015). A framework to reduce infectious disease risk from urban poultry in the United States. Publ. Health Rep..

[20] Brinkley C., Kingsley J. (2018). A chicken in every backyard: urban poultry needs more regulation to protect human and animal health. Convers..

[21] Ayala A.J. (2022). Risky business in Georgia's wild birds: contact rates between wild birds and backyard chickens is influenced by supplemental feed. Epidemiol. Infect..

[22] Greening S.S., Gates M.C. (2023). A cross- sectional study of ownership of backyard poultry in two areas of Palmerston North, New Zealand. N. Z. Vet. J..

[23] Paphitis J. (2023). Backyard chickens — a cross-sectional survey of current and prospective backyard chicken owners in Ontario (2019–2021). Can. Vet. J..

[24] ChickenGuard (2019). Chicken keeping on the rise in Australia.

[25] Poultry Extension (2023). Developing regulations for keeping urban chickens.

[26] (2022). City of Vancouver. Backyard Hens.

[27] (2023). City of Kitchener. Backyard Chickens.

[28] (2023). City of Toronto.

[29] Poultry Australia (2023). https://www.poultryaustralia.com.au/keeping-poultry.

[30] Citizens Advice Bureau (2023). Do I need to get permission to keep chickens In my back yard?.

[31] WHO (2023). Cumulative Number of Confirmed Human Cases for Avian Influenza A(H5N1) Reported to WHO, 2003-2023.

[32] Cristalli A., Capua I. (2007). Practical problems in controlling H5N1 high pathogenicity avian influenza at village level in Vietnam and introduction of biosecurity measures. Avian Dis..

[33] Van Kerkhove M.D. (2009). Changes in poultry handling behavior and poultry mortality reporting among rural Cambodians in areas affected by HPAI/H5N1. PLoS One.

[34] Sultana R. (2012). Bangladeshi backyard poultry raisers' perceptions and practices related to zoonotic transmission of avian influenza. J. Inf. Dev. Ctries.

[35] Conan A. (2012). Biosecurity measures for backyard poultry in developing countries. BMC Vet. Res..

[36] Sims L.D. (2013). Intervention strategies to reduce the risk of zoonotic infection with avian influenza viruses. Infl. Other Respir Vir..

[37] Rimi N.A. (2016). Understanding the failure of a behavior change intervention to reduce risk behaviors for avian influenza transmission among backyard poultry raisers in rural Bangladesh: a focused ethnography. BMC Publ. Health.

[38] Rimi N.A. (2018). Where backyard poultry raisers seek care for sick poultry. BMC Publ. Health.

[39] Tenzin T. (2017). Biosecurity survey in relation to the risk of HPAI outbreaks in backyard poultry holdings in Thimphu city area, Bhutan. BMC Vet. Res..

[40] Barennes H. (2010). Paradoxical risk perception and behaviours related to Avian Flu outbreak and education campaign, Laos. BMC Inf. Dis..

[41] Manabe T. (2011). Impact of educational intervention concerning awareness and behaviours relating to avian influenza (H5N1) in a high-risk population in Vietnam. PLoS One.

[42] Conan A A. (2013). A community-based education trial to improve backyard poultry biosecurity in rural Cambodia. Acta Trop..

[43] Michie S. (2011). The behaviour change wheel. Implement. Sci..

[44] Michie S. (2013). The behavior change technique taxonomy (v1) of 93 hierarchically clustered techniques. Ann. Behav. Med..

[45] Dreibelbis R. (2013). The integrated behavioural model for water, sanitation, and Hygiene. BMC Publ. Health.

[46] Sesan T. (2018). Toilet training: what can the cookstove sector learn from improved sanitation promotion?. Int. J. Environ. Health Res..

[47] Clasen T., Smith K.R. (2019). ‘Let the “A” in WASH stand for air. Environ. Health Perspect..

[48] Jewitt S. (2022). Domesticating cleaner cookstoves for improved respiratory health: using approaches from the sanitation sector to explore the adoption and sustained use of improved cooking technologies in Nepal. Soc. Sci. Med..

[49] Curtis V. (2019). Behaviour settings theory applied to domestic water use in Nigeria. Soc. Sci. Med..

[50] Shove E. (2012).

[51] E. McClaughlin, et al. UK Flockdown: Exploring the Knowledge, Attitudes and Practices of Backyard Poultry Keepers Surrounding Highly Pathogenic Avian Influenza (HPAI). In Review.

[52] McClaughlin E. (2023). ‘The reception of public health messages during the COVID-19 pandemic.’. Appl. Corp. Ling..

[53] Hill A. (2019). ‘Quantifying the spatial risk of Avian Influenza introduction into British poultry by wild birds.’. Sci. Rep..

[54] Defra (2021). https://www.gov.uk/guidance/avian-influenza-bird-flu.

[55] UK Government (2022). https://www.gov.uk/government/news/bird-flu-latest-situation-avian-influenza-prevention-zone-declared-across-great-britain.

[56] Diefenbacher S. (2022). Differences in observed and self-reported compliance with ‘Five Moments for Hand Hygiene’ as a function of the empathy of healthcare workers. J. Hosp. Infect..

[57] Sutherland L.A. (2013). Considering the source. J. Environ. Manag..

[58] BBC (2011). https://www.bbc.co.uk/news/uk-england-12483017.

[59] BBC (2021). https://www.bbc.co.uk/news/uk-england-bristol-58255378.

[60] Government of Scotland. Protecting Poultry Health and Preventing Disease. (No date). Available at: https://www.gov.scot/binaries/content/documents/govscot/publications/advice-and-guidance/2018/10/avian-influenza-bird-flu/documents/protecting-poultry-health-and-preventing-disease/protecting-poultry-health-and-preventing-disease/govscot%3Adocument/Protecting%2Bpoultry%2Bhealth%2Band%2Bpreventing%2Bdisease.pdf (accessed 10/06/22).

[61] Defra, Welsh Government, Scottish Government, NFU, BHWT, RSPCA, The Poultry Club of Great Britain, Do you keep chickens, ducks, geese…? Help protect your birds from the risk of #birdflu. (No date) Available at: https://gov.wales/sites/default/files/publications/2021-01/avian-influenza-bird-flu-help-protect-your-birds.pdf (accessed 03-06-22).

[62] Welsh Government (2017). https://gov.wales/sites/default/files/publications/2017-11/avian-influenza-bird-flu-advice-for-keepers-of-small-flocks.pdf.

[63] APHA (2021). https://www.youtube.com/watch?v=t3Aq0iCaKS0.

[64] Karabozhilova I. (2012). Backyard chicken keeping in the Greater London Urban Area: welfare status, biosecurity and disease control issues. Br. Poultry Sci..

[65] Burns T.E. (2011). Preliminary investigation of bird and human movements and disease- management practices in noncommercial poultry flocks in south- western British Columbia. Avian Dis..

[66] Burns T.E. (2012). Perspective of an underrepresented stakeholder group, backyard flock owners, on poultry health and avian influenza control. J. Risk Res..

[67] Lockhart C.Y. (2010). A cross- sectional study of ownership of backyard poultry in two areas of Palmerston North, New Zealand. N. Z. Vet. J..

