# Supplementary material for: Exploring the responses of smallscale poultry keepers to avian influenza regulations and guidance in the United Kingdom, with recommendations for improved biosecurity messaging
Source: Heliyon. 2023 Aug 17;9(9):e19211. doi: 10.1016/j.heliyon.2023.e19211 (PMC10470266; doi:10.1016/j.heliyon.2023.e19211)
Supplement: Multimedia component 1 [file mmc1.pdf]

Exploring the Responses of Smallscale Poultry Keepers to Avian Influenza Regulations and Guidance in the United Kingdom, with Recommendations for Improved Biosecurity Messaging.

Supplementary file 1: Survey information and flow diagram for backyard keepers' survey

The University of Nottingham
UNITED KINGDOM · CHINA · MALAYSIA

Avian Influenza Poultry Keeper Survey

This survey is being conducted by the School of Veterinary Medicine and Science at the University of Nottingham. The study has ethical approval from the School's clinical ethics panel. We are seeking to understand the impacts of the messaging around Avian Influenza outbreaks in the UK on small scale poultry producers. All responses to the survey will be held on a secure database at the University of Nottingham and will be anonymised. The collated results of the survey may be published in academic journals or shared with government, veterinary or poultry industry bodies but will not be used for commercial purposes. Individual responses will not be identifiable in these reports. If you would like to receive a copy of the results of the survey you can provide an email address for this to be sent to. If you wish for your responses to be removed or to view the data held from your responses at any stage please contact the researchers involved in this survey. For any further information on the study please contact:

Sol Elliott svyse2@exmail.nottingham.ac.uk
Associate Professor Rachael Tarlinton rachael.tarlinton@nottingham.ac.uk
Clinical Associate Professor Michael Clark michael.clark2@nottingham.ac.uk

School of Veterinary Medicine and Science,
University of Nottingham,
Sutton Bonington Campus
Loughborough
LE12 5RD

1. *Please provide us with the first section of your postcode (e.g. LE12) so we can gauge an idea of where poultry flocks are located.

2. *What type of birds do you keep? Please tick all that apply: Chickens, Ducks, Turkeys, Geese, Game birds, Other (please specify)

3. *How many birds do you have in your flock? 1-10, 11-20, 21-50, 51-100, 101-150, 151-199, 200+

4. *Where do you get your birds from? Purebreed – bought in, Purebreed – home reared, Mixed breed – bought in, Mixed breed – home reared, Rescue poultry, Other (please specify)

5. *How are your birds normally housed? Shed (no outdoor access), Coop and run; Garden/field (free range); Confined at night (coop and run), free range during the day; Other (Please specify)

6. *Please choose the type of shelter most similar to what you provide your birds with (If you previously answered, 'coop and run' please describe both coop and the run below). Tick all that apply: Wooden coop, Metal coop, Plastic coop, Portable coop, Fixed coop, Run: Concrete floor, Run: Soil floor, Run: Wooden frame and chicken wire, Run: Metal fences, Other (please specify)

7. *What kind of area would you say you keep your chickens in: Urban area – garden, Urban area – other, Semi-rural area – garden, Semi-rural area – other, Rural area – garden, Rural area – other

8. *Due to the nature of your bird housing, would it possible that your birds could have contact with any of the following birds, either wild or which don't belong to you? Please tick all that apply: No, Chickens, Ducks, Turkeys, Geese, Pigeons, Partridges, Quail, Guinea fowl

9. *Are you aware that it is a legal requirement for owners of flocks of 50+ birds (whether that is the same or different species of bird) to be signed up to the poultry register?

10. *Are you aware you can sign up to the poultry register voluntarily if you keep less than 50 birds or keep birds as pets, allowing APHA to contact you in the event of a disease outbreak (such as the recent Avian Influenza outbreak)?

11. *Have you heard of the recent outbreak (November 2021) of Avian Influenza and the control measures you need to implement as part of the mandatory housing order?

Yes → 12; No → 17

12. *Where did you get this information? Social media, Poultry register communication, Local vet, Internet, Other (please specify)

13. *Please specify which organisation was giving out this information on Avian Influenza: Local vets, DEFRA, APHA, Government, RSPCA, DAERA, NFU, BASC, British Hen Welfare Trust, Other (please specify):

14. *After receiving this information, what do you understand you need to do as part of the mandatory housing order?

15. *How easy were the adjustments to make, in order to comply with the housing measures? 1. Impossible, 2. Very difficult, 3. Okay, 4. Easy, 5. Very easy

16. *If you couldn't implement the housing measures, please tell us why. If this doesn't apply, please leave blank.

17. *What would make you suspect your birds had Avian Influenza?

18. *What would your next course of action be after suspecting a case in your flock?

19. *What are your views on the obligatory culling of all birds on the premises of a confirmed Avian Influenza case?

20. *If a vaccine were to become available for Avian Influenza which may mean that some of the control measures could differ from the current methods used (e.g. mandatory housing), would you vaccinate your chickens? If so, how much would you be willing to pay per dose, not including other veterinary fees? No; Yes, £0.01-2.50; Yes, £2.50-5; Yes, £6-10; Yes, £11-15 Yes, £15-20; Yes, £20+

21. *Would you administer the vaccine yourself (under veterinary advice) or would you prefer them to be vaccinated by a vet? Do it myself, feel confident in doing so; Do it myself, following training from my vet; Prefer if my vet did it
