# Supplementary material for: Exploring the responses of smallscale poultry keepers to avian influenza regulations and guidance in the United Kingdom, with recommendations for improved biosecurity messaging
Source: Heliyon. 2023 Aug 17;9(9):e19211. doi: 10.1016/j.heliyon.2023.e19211 (PMC10470266; doi:10.1016/j.heliyon.2023.e19211)
Supplement: Multimedia component 2 [file mmc2.docx]

**Exploring the Responses of Smallscale Poultry Keepers to Avian Influenza Regulations and Guidance in the United Kingdom, with Recommendations for Improved Biosecurity Messaging.**

**Supplementary file 2: Methodological Approach**

**Questionnaire survey participants**

Two UK-based poultry charities were contacted and agreed to distribute the survey: British Hen Welfare Trust and Fresh Start for Hens. The questionnaire survey was also advertised on social media and was targeted at a range of poultry keeping groups and organisations. Some of these groups focused more generally on ‘poultry’ while others were more specific such as ‘ducks ducks and ducks UK’ and ‘Quail Breeding in the UK’. A list of groups contacted and their membership numbers are provided in Table 1 below. Permission to post a link to the survey was requested from the group administrators. As the survey was advertised on social media, poultry keeping forums or email distribution lists which all require internet access, poultry keepers without access to the internet could not complete it. As the questions were only available in English this may have made the survey difficult for non-English-speaking poultry keepers to complete.

**Table 1. Membership size of the poultry groups that advertised the survey**

| Online group or forum | Membership |
| --- | --- |
| Backyard Ducks | 19,500 |
| British Hen Welfare Trust | 58,000 |
| Chicken & Poultry Keeping UK | 10,800 |
| Chicken and Poultry Keeping UK | 25,100 |
| Chicken Keepers UK | 19,000 |
| ducks ducks & ducks UK | 6,900 |
| Ducks UK | 3,200 |
| Ex Battery Hens Forum | 10,000 |
| Fresh Start for Hens | 43,000 |
| Keeping Hens and Poultry for Beginners | 10,400 |
| Omlet Chicken Keeping Community UK | 5,000 |
| Poultry UK | 11,100 |
| Quail Breeding in the UK | 5,500 |
| Raising Ducks | 107,500 |
| Silkies for pets, UK | 7,700 |
| Sustainable Chicken & Duck Keepers UK; | 9,400 |
| Midlands Poultry | 6,000 |
| The Hen House | 9,100 |
| Wing and a Prayer Rescuers | 2,400 |
| Avian Influenza aka “Bird flu” | 3,000 |

Information on the total number of smallscale keepers is hard to find as, with the exception of Northern Ireland, where all flocks must be registered with the Department of Agriculture, Environment and Rural Affairs (DAERA)^[[1]](#footnote-1)^ it is not compulsory for keepers of fewer than 50 birds to register their flocks with the Department for Environment, Food and Rural Affairs (Defra)^[[2]](#footnote-2)^ and the Animal and Plant Health Agency (APHA).^[[3]](#footnote-3)^ Nevertheless, a 2020 survey of chicken keepers estimated that there were 1,338,000 chicken owners in the UK [17] and that these numbers were on the rise. Assuming a target population of 1.5 million smallscale keepers, we exceeded our target sample of 400 (95% confidence) by some considerable margin. As Fig. 1 illustrates, we received responses from across the UK.

Noting the lack of definitional consensus for what constitutes a ‘backyard keeper’ [12], we elected to allow keepers to self-define as ‘small-scale’ for participation in this study. Over 96% of respondents reported keeping 50 or fewer birds. Following the GDPR principle of 'data minimization', whereby any personal data gathered must be 'adequate, relevant and limited to what is necessary in relation to the purposes for which they are processed' we did not collect demographic data on either the survey or workshop participants but information on their locations can be found in Fig. 1 and Table 3 of the main manuscript. No other personal information was sought although those respondents who wished to receive a copy of the survey results were given the opportunity to include their email address.

**Questionnaire format**

There were 21 questions in the online survey. Five of these (Q14 and Q16 through 19) invited open text responses and 16 were closed questions. The final version of the questionnaire including the skip logic can be found in Supplementary file 1. The findings reported in this study draw primarily on respondents’ answers to questions 14 and 16 with information from questions 11–13, 15 and 20 presented for context. A more comprehensive analysis of quantitative data from the survey forms the basis for a separate manuscript [51].

Respondents were asked to provide the first part of their postcode (Q1), information on the numbers, species and origin of the poultry kept (Q2–4) and the nature of their poultry set-ups (Q5–8). Questions 9–10 sought information on keepers’ knowledge of the Great Britain Poultry Register and Q11–16 asked if respondents were aware of the recent avian influenza outbreak, where they received information on it, what adjustments they needed to make, how difficult they found these and – if applicable, why they were unable to implement the housing measures. Questions 17–19 asked respondents to say what would make them suspect that their birds had avian influenza, what they would do if they suspected avian influenza and what they thought about obligatory culling. Questions 20–21 asked how much respondents would pay to vaccinate their birds (if a vaccine was available as an alternative control strategy) and whether they would administer this themselves or with assistance from a veterinarian.

The questionnaire was hosted on Microsoft Forms and prior to being made publicly available, it was piloted by 5 respondents with either practical experience of keeping poultry in backyard settings or expertise in veterinary medicine (or both). Feedback from the pilot resulted in adaptations to improve clarity and to make it easier for respondents to answer (e.g. the addition of tick box tables). The estimated time for completion stated on the information to respondents was 5-10 mins based on the results of the pilot study. The average time for completion over the 1559 respondents was eight minutes and 27 seconds.

**Participatory workshop.**

Question 23 of the survey invited respondents to provide their email address if they wished to receive a copy of the survey outcomes and summaries. A total of 637 survey respondents provided an email address and were sent a summary of the survey results. At the same time, they were informed of and invited to attend a workshop in July 2022. All participants who accepted the invitation to attend the workshop were accommodated with 21 attending in total (0.16% of those who expressed an interest in participating further in the study).

The workshop was held at the School of Veterinary Medicine and Science, University of Nottingham, as it is centrally located within the UK Midlands with good transport networks and it offered an opportunity for participants to interact with researchers with interests in backyard poultry and HPAI. The workshop took place on a Saturday to facilitate attendance. All participants who accepted the invitation were accommodated at the workshop and provided with refreshments and lunch.

Prior to attending, participants were invited to send photographs of their poultry keeping settings to aid discussion during the workshop. These were printed by the project team along with copies of HPAI-related guidance aimed at smallscale keepers plus government regulations and guidance associated with the 2021–1 declaration of an Avian Influenza Prevention Zone – including housing measures [9,54]. These materials were placed on three separate tables during the workshop along with different coloured post-its, drawing/writing materials, bird and farm-themed stickers, plain A4 paper and flip chart sheets.

The first part of the workshop involved presentations that provided a general overview of HPAI, the spatial distribution of cases in the UK, summaries of the survey findings and an introduction to the workshop activities. Participants were then invited to divide themselves roughly equally into three discussion groups (W1–3) sitting at 3 separate tables where conversations and activities were facilitated by at least two members of the project team. All activities had been planned in advance by the team but to facilitate synchronous discussions, a summary with suggested timings for each activity was provided to all team members and participants. All of the authors attended and participated in the workshop as facilitators, helping to explain the different activities and guide the discussions.

The workshop activities were organised into three sessions; each of which involved a range of activities. The discussions and activities included topics and activities that generated co-produced knowledge about:

- aspects of official HPAI-related guidance that were confusing or not widely implemented
- suggestions for clarifying areas of confusion or lack of awareness
- aspects of the housing measures that smallscale keepers found hard to comply with
- inexpensive, easy-to-implement measures to adapt poultry-keeping settings to increase biosecurity and comply with housing measures.
- more effective approaches for communicating the regulations to smallscale keepers
- possible alternative approaches to housing measures (including vaccination) and their likely acceptability to smallscale keepers

***Workshop Session* *1*** focused on understandings of avian influenza housing measures and prevention regulations. The activities in this session were divided into 3 tasks.

***Workshop Session 1 Task 1***

- Participants were asked to introduce themselves to other group members outlining what types (and how many) poultry they kept.
- They were then encouraged to write their thoughts about the housing measures in place from 29 November 2021 to 2 May 2022 (using orange post-its) and the ‘protection zone’ measures from 2 May 2022 (using green post-its) and discuss any areas of confusion.
- They were also asked to note down the key points that they remembered of the housing measure regulations (using orange post-its) and protection zone measures (use green post-its).

***Workshop Session 1 Task 2***

- Participants and facilitators grouped post-its with similar points and compared them with copies of the regulations (available on the table) to identify regulations that were well understood/implemented and those that were not.
- Participants were then asked to highlight regulations that they found unclear or confusing, or were unaware of.
- Next, participants were asked to think about their own poultry keeping settings and consult photographs that had been sent in advance of the workshop to highlight inexpensive, easy-to-implement measures that complied with the regulations and could be adopted more widely.

***Workshop Session 1 Task 3***

- Participants were asked whether they had any experience of being within 3km Protection zones or 10km Surveillance zones and to share these if so, noting down what additional restrictions applied in 3km Protection zones (on pink post-its) and in 10km Surveillance zones (on yellow post-its)
- They were then asked to identify anything about the 3km and 10km zone regulations that they found unclear.

***Workshop Session 2*** focused on improving the clarity of HPAI-related housing measures and prevention regulations. There were two tasks in this session, the second of which involved the co-production of clearer messaging for smallscale poultry keepers**.**

***Workshop Session 2: Task 1***

- Participants were asked to build on previous activities to identify which regulations were well-understood and which were confusing.
- They were also asked to make notes or put post-its against the regulations that they found easy to implement and that that they found difficult.

***Workshop Session 2: Task 2***

- Participants were then asked to suggest different forms of wording or diagrams and informatics that might help to clarify the regulations.
- Next they were asked about where they obtained information on the housing measures and protection zone requirements, whether this was easy to find and whether they trusted the information source they used.
- Lastly, they were asked for suggestions on what could be done to make information on avian influenza easier to find and understand.

***Workshop Session 3*** sought to elicit respondents’ views on possible alternatives to the measures taken to prevent the spread of avian influenza and possible barriers to their adoption by backyard keepers.

***Workshop Session 3: Task 1:***

- Participants were asked to discuss and note down their thoughts on what alternative approaches to ‘flockdown’ could be used in future.
- They were also asked to identify the main advantages and disadvantages of these alternatives.

***Workshop Session 3: Task 2:***

- In this activity, participants were asked to discuss their thoughts on vaccination as a possible alternative approach (if permitted).
- In particular, they were asked about the advantages and disadvantages of vaccination for backyard keepers compared to the approaches they identified in task 1.

The workshop ended with a question-and-answer session where participants had the opportunity to discuss a range of poultry-related issues with the research team.

**Data analysis**

Responses to the closed survey questions were checked for completion and for the presence of a UK postcode. One response with a non-UK postcode was removed along with two incomplete responses leaving a total of 1556 complete responses. Summary statistical analysis was undertaken on these responses in Microsoft Excel (v2303). Information on the workshop poultry flock demographics can be found in Table 3 of the manuscript.

Qualitative data from the three workshop discussion groups and survey questions 14 and 16 were collated by the lead author and reviewed to get a sense of the key themes emerging. Manual inductive coding of participants’ comments, facilitators’ notes and other outputs (e.g. annotated drawings and comments on the regulations and poultry setup photographs) was then undertaken by the lead author to generate initial codes from the data. During the coding process, regular discussions were held with the co-authors regarding any points that were ambiguous or unclear. Subsequent rounds of coding were undertaken by the lead author with input from co-authors who acted as facilitators on the different tables to identify and agree sub-themes and additional codes.

These codes were subsequently mapped onto the IBM-WASH framework to ascertain whether this would provide a useful way to identify key barriers and enablers to compliance with HPAI-related regulations and guidance. It was agreed that the codes aligned well with the different levels of the ‘context’ and ‘psychosocial’ domains but had limited relevance to the ‘technology’ domain. We therefore decided to replace this dimension with one focusing on co-produced recommendations and suggestions that could potentially be implemented at different levels. The findings are presented in the main manuscript according to this revised IBM-WASH model structure (Table 4).

**Dissemination of findings from the research**

Presentations drawing on the results of the survey were made to the British Veterinary Poultry Association (BVPA) and the Poultry Health and Welfare Group (PHWG). BVPA has 224 members who are poultry veterinary surgeons working in private poultry practice, academia, industry and government. The PHWG is a combined poultry industry group made up of officers/committee members of the British Poultry Council (BPC), the British Egg Industry Council (BEIC), National Farmers Union (NFU), Game Farmers Association (GFA) and the BVPA. A further presentation was made at the Royal College of Veterinary Surgeons Fellowship Day which was attended by veterinarians (including poultry specialists) and representatives from DEFRA.

1. DAERA is a government department in the Northern Ireland Executive, the devolved administration for Northern Ireland. It has responsibility for food, farming, environmental, fisheries, forestry and sustainability policy and the development of the rural sector in Northern Ireland [↑](#footnote-ref-1)
2. Defra is a UK government department with responsibility for improving and protecting the environment and supporting the food, farming and fishing industries. [↑](#footnote-ref-2)
3. The Animal and Plant Health Agency (APHA) is an executive agency of the Department for Environment, Food & Rural Affairs, and also works on behalf of the Scottish Government and Welsh Government [↑](#footnote-ref-3)
